# TLR2 Regulates Mast Cell IL-6 and IL-13 Production During *Listeria monocytogenes* Infection

**DOI:** 10.3389/fimmu.2021.650779

**Published:** 2021-06-14

**Authors:** Rodolfo Soria-Castro, Ángel R. Alfaro-Doblado, Gloria Rodríguez-López, Marcia Campillo-Navarro, Yatsiri G. Meneses-Preza, Adrian Galán-Salinas, Violeta Alvarez-Jimenez, Juan C. Yam-Puc, Rosario Munguía-Fuentes, Adriana Domínguez-Flores, Sergio Estrada-Parra, Sonia M. Pérez-Tapia, Alma D. Chávez-Blanco, Rommel Chacón-Salinas

**Affiliations:** ^1^ Departamento de Inmunología, Escuela Nacional de Ciencias Biológicas, Instituto Politécnico Nacional (ENCB-IPN), Mexico City, Mexico; ^2^ Research Coordination, Centro Médico Nacional 20 de Noviembre, Instituto de Seguridad y Servicios Sociales de los Trabajadores del Estado (ISSSTE), Mexico City, Mexico; ^3^ Unidad de Citometría de Flujo, Lab de Biología Molecular y Bioseguridad Nivel 3, Centro Médico Naval, Secretaría de Marina (SEMAR), Mexico City, Mexico; ^4^ Institute of Immunology and Immunotherapy, College of Medical and Dental Sciences, University of Birmingham, Birmingham, United Kingdom; ^5^ Departamento de Ciencias Básicas, Unidad Profesional Interdisciplinaria en Ingeniería y Tecnologías Avanzadas, Instituto Politécnico Nacional (UPIITA-IPN), Mexico City, Mexico; ^6^ Unidad de Desarrollo e Investigación en Bioprocesos (UDIBI), Escuela Nacional de Ciencias Biológicas, Instituto Politécnico Nacional (ENCB-IPN), Mexico City, Mexico; ^7^ Subdirección de Investigación Básica, Instituto Nacional de Cancerología (INCan), México City, Mexico

**Keywords:** mast cell, *Listeria monocytogenes*, toll like receptor-2, IL-6, IL-13, p38, MAPK, LLO

## Abstract

*Listeria monocytogenes* (L.m) is efficiently controlled by several cells of the innate immunity, including the Mast Cell (MC). MC is activated by L.m inducing its degranulation, cytokine production and microbicidal mechanisms. TLR2 is required for the optimal control of L.m infection by different cells of the immune system. However, little is known about the MC receptors involved in recognizing this bacterium and whether these interactions mediate MC activation. In this study, we analyzed whether TLR2 is involved in mediating different MC activation responses during L.m infection. We found that despite MC were infected with L.m, they were able to clear the bacterial load. In addition, MC degranulated and produced ROS, TNF-α, IL-1β, IL-6, IL-13 and MCP-1 in response to bacterial infection. Interestingly, L.m induced the activation of signaling proteins: ERK, p38 and NF-κB. When TLR2 was blocked, L.m endocytosis, bactericidal activity, ROS production and mast cell degranulation were not affected. Interestingly, only IL-6 and IL-13 production were affected when TLR2 was inhibited in response to L.m infection. Furthermore, p38 activation depended on TLR2, but not ERK or NF-κB activation. These results indicate that TLR2 mediates only some MC activation pathways during L.m infection, mainly those related to IL-6 and IL-13 production.

## Introduction

Classically, mast cells (MC) are associated with type I hypersensitivity reactions (1). However, growing evidence has placed them as initiators of the inflammatory process against several infectious agents, including bacteria (2)⁠. Due to their strategic location in mucosal epithelia, skin, and connective tissue, they can respond immediately to the signals derived from mutualistic and pathogenic bacteria, adapting their response accordingly to maintain host homeostasis (3)⁠. In this way, MC are provided with at least one member of each of the Pattern Recognition Receptor (PRR) families. These include Toll-Like Receptors (TLR), C-type Lectin Receptors (CLR), NOD-Like Receptors (NLRs), RIG-Like Receptors (RLRs), and Scavenger Receptors (4). When MC are activated, they release many preformed mediators found in their granules, through cell degranulation. Furthermore, they can also synthesize *de novo* molecules such as inflammatory mediators derived from arachidonic acid, reactive oxygen species (ROS) as well as cytokines and chemokines (5). Several studies have shown that MC are involved in the immune response to pathogenic bacteria, including: *Pseudomonas aeruginosa* (6), *Klebsiella pneumoniae* (7), *Staphylococcus aureus* (8), *Mycobacterium tuberculosis* (9) and *Listeria monocytogenes* (L.m) (10)⁠, to mention only a few.

L.m is a Gram-positive, facultative intracellular bacteria and the causal agent of listeriosis, a foodborne disease with a high mortality rate (11)⁠. L.m presents tropism towards the gravid uterus and central nervous system (CNS), contributing to the most severe clinical manifestations (12). Although, these severe cases are rare in immunocompetent individuals, they increase in case of immunosuppression or immunodeficiencies (11)⁠. This implies that host immune response mechanisms are crucial to the containment of the bacteria, and its alteration can increase susceptibility to the infection (13).

Cells of the innate immune response play a crucial role in containing L.m infection, notably, macrophages, dendritic cells, neutrophils, NK cells and MC (14, 15)⁠. In experimental murine listeriosis models, MC have been shown to initiate the effector response against this bacterium, promoting recruitment of neutrophils and macrophages to the site of infection (10, 15). Furthermore, MC response to L.m includes intracellular infection (16), degranulation (16, 17), ROS production (18) and different cytokines and chemokines (15, 16)⁠. However, it is unclear which receptors mediates L.m activation by MC.

TLR2 is a transmembrane type I receptor, which contains a cytoplasmic TIR domain as well as an extracellular domain with leucine-rich repeats (19). TLR2 ligands include: Diacil-lipopeptides, lipoarabinomannan, lipoproteins, lipoteichoic acid (LTA), peptidoglycan (PGN), porins, phospholipomannan and zymosan (20). Once TRL2 binds to its ligand, it is dimerized with either TLR1 or TLR6 (21). When this occurs, TIR domain recruits the adaptive molecules TIRAP and MyD88 that lead to the activation of the transcription factor NF-κB and the mitogen-activated protein kinases (MAPK) that activate the transcription factor AP-1 (21, 22)⁠. Additionally, TLR2 activates the P13K-AKT signaling pathway (22)

Activation of TLR2 promotes diverse cellular functions such as phagocytic activity (23)⁠, bactericidal activity (24), ROS production (25), degranulation (26) and cytokine production (27)⁠. In addition, TLR2-deficient mice are more susceptible to L.m infection than wild type mice, which is consistent with poor control of the bacterial load on target organs (28)⁠. In addition, MyD88-deficient MC produce less IL-6 and MCP-1 in response to L.m (16)⁠. Considering that MC express TLR2 (29), we decided to dissect the activation mechanisms that are regulated by TLR2 during mast cell activation by *Listeria monocytogenes* infection.

## Material and Methods

### Bacteria Culture

L.m strain 1778+H 1b (ATCC 43249, USA, Manassas, VA, USA) was grown in brain heart infusion broth (BHI, BD-Difco, USA) for 18 h at 37°C with constant shaking at 112 xg. Bacterial cultures were washed with Hanks Balanced Saline Solution (HBSS) (Life Technologies, USA) and bacterial pellets resuspended in RPMI-1640 Glutamax (Life Technologies, USA) supplemented with 40% Fetal Bovine Serum (Life Technologies, USA) and frozen at -70 °C until use. Aliquots of L.m were serially diluted and plated in BHI agar at 37°C for 18-24 h. Bacterial numbers were determined by counting Colony-Forming Units (CFU).

### Mast Cells

Bone Marrow-derived Mast Cell (BMMC) was obtained following the protocol described by (30). Briefly, bone marrow cells were obtained from femurs and tibias of 6-8-week-old C57BL/6 female mice. Cells were maintained in RPMI-1640 supplemented with 10% FBS, 5 μM β-mercaptoethanol (Life Technologies, USA) and 2% antibiotic and antimycotic (Sigma, USA) (complete RPMI 1640 medium) plus 10 ng/mL of murine recombinant IL-3 (Peprotech, USA) and 10 ng/mL of murine recombinant stem cell factor (Peprotech, USA)). Non-adherent cells were transferred to fresh culture medium twice a week for 6−9 weeks. The purity of BMMC was ≥90% assessed by flow cytometry after staining of CD117 (clone: 2B8, BioLegend, USA; 0.25 μg/mL) and FcϵRI (clone: MAR-1, BioLegend, USA; 0.16 μg/mL) (**Supplementary Figure 1A**).

Peritoneum-derived mast cells (PMC), cells were obtained from peritoneal cavity of mice and cultured in complete RPMI-1640 medium plus IL-3 (30 ng/mL) and SCF (20 ng/mL). Non-adherent cells were transferred to fresh culture medium twice a week for 3−4 weeks. The purity of PMC was ≥90% assessed by flow cytometry (**Supplementary Figure 1C**).

All experiments followed institutional biosecurity and safety procedures. All animal experiments were approved by the Research Ethics Committee of the ENCB, IPN (ZOO-016-2019).

### Toluidine Blue Staining

2x10^5^ BMMC or 2x10^5^ PMC/0.25 mL of RPMI-1640 supplemented with 10% FBS and 5μM β-mercaptoethanol (complete medium) were plated in cytospin chambers, and then stained with toluidine blue for 10 minutes. Finally, slides were air-dried and mounted with Entellan resin (Merck Millipore, USA) under a coverslip. Images were captured with a digital camera attached to a brightfield microscope (Zeiss Primo Star, Germany), and analyzed with Micro capture v7.9 software (**Supplementary Figure 1B, D**).

### Viability Mast Cell Assay

2.5X10^5^ BMMC/0.25 mL of complete medium were stimulated with L.m at different MOI (1:1, 10:1, 100:1) or stimulated with LLO (125, 250, 500, 1000 ng/mL) for 24 h. Then cells were washed with 1 mL of Annexin V binding Buffer 1X (Invitrogen, USA) and stained with 1 μg/mL Annexin V (BioLegend, USA) and 0.5 μg/mL propidium iodide (eBioscience, USA). After staining, cell viability was measured by flow cytometry (**Supplementary Figure 2**).

### Degranulation Assay

The degranulation assay was carried out as described previously (31). Briefly, 2x10^5^ BMMC/0.25 mL of complete medium were incubated with L.m at different MOI for 90 minutes. Then were washed and stained with anti-CD107a (clone: 1D4B Biolegend, USA; 0.25 μg/mL) and anti-FcϵRI. Staining was measured by flow cytometry.

β-Hexosaminidase release was performed as follows: 2x10^5^ BMMC/0.25 mL of HEPES-Tyrode Buffer (HBT) (130 mM NaCl, 5.5 mM glucose, 2.7 mM KCl, 1.0 mM CaCl_2_ 2 H_2_O, 0.1% [wt/vol] Bovine Serum Albumin (BSA), 12 mM HEPES, 0.45 mM NaH_2_PO_4_ 1H_2_O, pH 7.2) were stimulated with L.m at different MOI or stimulated with PMA (125 nM) plus Ionomicin (10 μM) for 90 minutes at 37°C. The supernatants were then recovered, and the cell pellet was lysed with 200 μL of 0.2% Triton X-100 in HBT. Both supernatants and cell lysates were incubated with 4-methylumbelliferyl N-acetyl-β-D-glucosaminide (Sigma-Aldrich, USA; 1 mM in 200 mM Na Citrate Buffer pH 4.5) for 2 h at 37°C. The enzyme reaction was stopped by the addition of 100 μL of 200 mM Tris base, pH 10.7. The samples were analyzed in a fluorescence plate reader (SpectraMax M, USA) using excitation 356 nm and emission 450 nm. The percentage of release of β-Hexosaminidase is calculated using the formula: $\%\;Release=\left[\frac{supernatan}{\left(supernatan+cellpellet\right)}\right]\times 100$.

### Superoxide Anion$\left({\text{O}}_{2}^{-}\right)$ Production

2x10^5^ BMMC were cultured in 0.25 mL of RPMI-1640 without phenol red (Life Technologies, USA) and then, stimulated with L.m at different MOI for 120 minutes. In the last 15 minutes of incubation 25 μL of a solution of p-nitro blue tetrazolium (NBT) (Sigma-Aldrich, USA) at 1 mg/mL were added. Then, cells were washed with PBS 1X (Life Technologies, USA) and fixed with absolute methanol. The formazan precipitates were dissolved by adding 54 μL of potassium hydroxide (KOH) 2mM and 46 μL of Dimethyl sulfoxide (DMSO) (Sigma-Aldrich, USA). The samples were read at a wavelength of 620 nm in a plate reader (Multiskan EX, Thermo Scientific, USA).

### Bacterial Load Assay

2x10^5^ BMMC/0.25 mL of complete medium were incubated with L.m at different MOI for 2 h. At the end of incubation, HBSS supplemented with ampicillin at 200 ng/mL was added for 1 h. Afterwards, cells were washed and then transferred to complete medium supplemented with ampicillin at 200 ng/mL for 0 and 24 h. Then, cells were washed with HBSS and then lysed with sterile distilled water. Cell suspension were homogenized, and serial decimal dilutions were prepared in saline solution. Afterwards, 20μL of each dilution were plated on BHI agar at 37°C for 48 h, bacterial numbers were determined by counting CFU.

### Cytokines Quantification

2x10^5^ BMMC/0.25 mL of complete medium were stimulated with L.m at different MOI or stimulated with Recombinant Listeriolysin-O (LLO; RayBiotech, USA) at different concentration (125, 250, 500 and 1000 ng/mL) for 24h. Then, supernatants were collected for the detection of TNF-α, IL-1β, IL-6, MCP-1 (Biolegend, San Diego, CA., USA) and IL-13 (eBioscience, USA) by ELISA according to manufacturer’s instructions.

### Evaluation of Phosphorylated Proteins

2x10^5^ BMMC/0.25 mL of complete medium were stimulated with L.m MOI 100:1 for 15 minutes to evaluate p-ERK 1/2, 30 minutes for p-p65 and 60 minutes for p-p38. Then, the cells were preserved with 250 μL of Fixation buffer (BD-Bioscience, USA) for 10 min/37°C. Subsequently, were washed with 1 mL of Stain Buffer (BD-Bioscience, USA) and permeabilized with 1mL of 0.5x Perm buffer IV (BD-Bioscience, USA) for 15 min at room temperature (RT) and protected from light. Then, the cells were washed with 1 mL of Stain Buffer. After blocking with 0.015 μg of anti-CD16/32 (Mouse BD Fc Block™, clone: 2.4G2. BD-Biosciences, USA) cells were stained with antibodies to p-ERK 1/2-PE (clone: 20A; 4 μL per tube), p-p65-PE (clone: K10-895.12.50; 4 μL per tube), p-p38-PE (Clone: 36/p38; 4 μL per tube) or isotype controls (Clone: MOPC-21; Mouse IgG_1_, κ-PE or Clone: MCP-11; Mouse IgG_2b_, κ-PE). (All from BD-Biosciences, USA) for 60 min at RT and protected from light. Finally, the cells were washed and resuspended in 0.15 mL of Stain Buffer and analyzed by flow cytometry. The times chosen for the detection of each phosphorylated protein were selected based on kinetic assay (**Supplementary Figure 4**).

### Expression of TLR2

2x10^5^ BMMC/0.1 mL of PBS 1X were marked with anti-TLR2-biotin (clone: 6C2, eBioscience, USA; 1 μg/0.1 mL) or isotype control-biotin (clone: eB149/10H5, eBioscience, USA; Rat IgG_2b_, κ-biotin; 1 μg/0.1 mL) and stained with streptavidin-APC (BD-Bioscience, USA; 0.02 μg/mL), prior blocking with anti-CD16/32. Staining was measured by flow cytometry.

### TLR2 Blocking Assays

2x10^5^ BMMC/0.25 mL of complete medium were pre-incubated 30 minutes with 200 ng/mL of anti-TLR2 (clone: C9A12; IgG2a) or 200 ng/mL of isotype control (clone: T9C6; 1gG2a), both from Invivogen, USA. Afterwards, cells were stimulated with L.m MOI 100:1 and the bacterial load, ${\text{O}}_{2}^{-}$ production, degranulation, cytokines production and evaluation of phosphorylated proteins assays were performed as indicated above. The bacterial load was analyzed as endocytic activity and bactericidal activity, according to the following formula: endocytic activity = (CFU at 0 h/MOI added) *100 (%). Bactericidal activity = (100-(CFU at 24 h/CFU at 0 h) *100) (%) (32). To evaluate the efficiency of antibody-mediated TLR2 inhibition, 2x10^5^ BMMC/0.25 mL were pre-incubated with different concentrations of anti-TLR2 (12.5, 25, 50, 100 y 200 ng/mL) for 30 min and then stimulated with the TLR2 agonist: *Staphylococcus aureus* peptidoglycan (PGN) (Sigma-Aldrich, USA) at 10 μg/mL for 24 h. Then, supernatants were collected for the detection of IL-6 by ELISA (**Supplementary Figure 5**).

### Flow Cytometry

All cell samples stained with fluorochrome-conjugated antibodies were acquired using FACSAria Fusion (BD Biosciences, USA) and analyzed with FlowJo software version 6.0 (FlowJo, LLC). Cell debris and doublets were excluded from the analysis.

### Statistical Analysis

All statistical analyses were performed with SigmaPlot software version 14.0, from Systat Software, Inc., San Jose California USA, www.systatsoftware.com. Data normality was assessed by Kolmogorov-Smirnov with Lilliefors correction. Variables that followed normal distribution were plotted as mean ± standard error mean (s.e.m), represented as bars and analyzed with one way-analysis of variance (ANOVA) with Student-Newman-Keuls (SNK) post-hoc. While variables that did not follow normal distribution, semi-quantitative variables, normalized variables or percentages, were plotted as median + range, represented as boxes and analyzed with the Mann-Whitney test (comparisons between two groups) or Kruskal-Wallis test (comparisons between more than two groups). A value of p < 0.05 was considered to be significant.

## Results

### Mast Cell Activation Concurs With the Control of *Listeria monocytogenes* Infection

**Figure 1:**
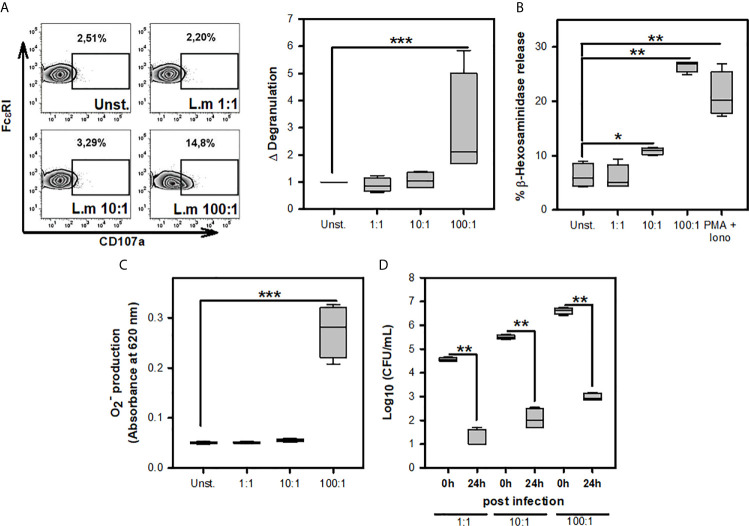
Mast cell activation in response to *Listeria monocytogenes* infection. **(A)** 2.5X10^5^ BMMC/0.25 mL of complete medium were stimulated with L.m for 90 minutes at the MOI indicated. BMMC degranulation was determined by flow cytometry. Left panel shows representative zebra-plots. Right panel shows the fold change in the percentage of FcϵRI^+^/CD107a^+^ cells with respect to unstimulated MC. (sum of 4 independent experiments, n=4 per group; ***p<0.001; Kruskal-Wallis test). **(B)** 2.5X10^5^ BMMC/0.25 mL of complete medium were stimulated with L.m at the MOI indicated or PMA (125 nM) plus Ionomycin (Iono; 10 μM) for 90 minutes. BMMC degranulation was determined through beta-hexosaminidase release as indicated in Material and methods. (sum of 4 independent experiments, n=4 per group; *p<0.05, **p<0.01 ***p<0.001; Kruskal-Wallis test). **(C)** 2.5X10^5^ BMMC/0.25 mL of RPMI-1640 without phenol red were stimulated with L.m for 2 h at the MOI indicated. Afterwards, the ${\text{O}}_{2}^{-}$determination was performed by the NBT reduction assay. (sum of 4 independent experiments, n=4 per group; ***p<0.001; Kruskal-Wallis test). **(D)** 2.5X10^5^ BMMC/0.25 mL of complete medium were infected with L.m for 2 h at the MOI indicated. Intracellular bacteria were quantified by CFU assay as indicated in Material and methods. (sum of 4 independent experiments, n=4 per group; **p<0.01; Mann-Whitney test).

Previous studies have noticed that MC can respond to L.m infection through degranulation, ROS production, internalization, and clearance of this bacterium (16–18). To corroborate the presence of these MC activation mechanisms during L.m infection, we incubated BMMC with different MOI of this bacterium to determine degranulation either through the surface expression of CD107a (**Figure 1A**) or β-hexosaminidase release (**Figure 1B**), ROS production with detection of ${\text{O}}_{2}^{-}$ (**Figure 1C**) and internalization and clearance of L.m by evaluating intracellular CFU (**Figure 1D**). We found that degranulation and ${\text{O}}_{2}^{-}$ production from BMMC occurred using L.m MOI 100:1 (**Figures 1A–C**). Also, we detected viable bacteria within BMMC at 0-hour post-infection (hpi) with the different MOI of L.m tested; however, we recovered small amounts of this bacterium at 24h, corresponding to a reduction of more than 3 logarithms for any MOI tested (**Figure 1D**). These results indicate that BMMC degranulate and produce ROS with high amounts of L.m and that BMMC are capable of internalizing and controlling L.m infection.

### *Listeria monocytogenes* Induces Mast Cell Cytokine and Chemokine Release

**Figure 2:**
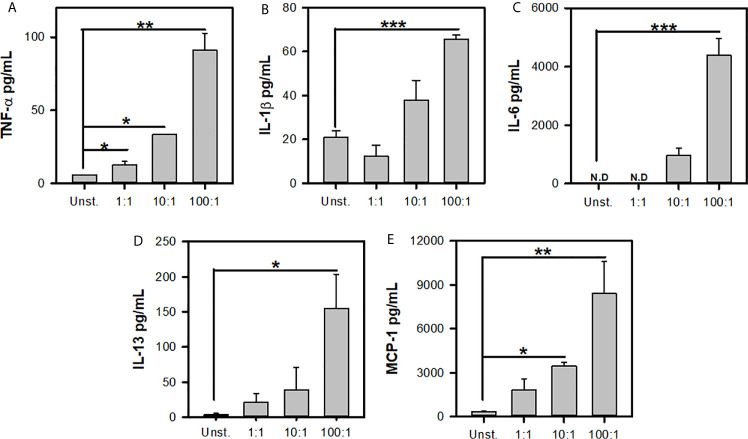
*Listeria monocytogenes* induces *de novo* synthesis of cytokines by mast cells. 2.5x10^5^ BMMC/0.25 mL of complete medium were stimulated with L.m for 24 h at the MOI indicated. Mediator levels were evaluated in culture supernatants by ELISA. **(A)** TNF-α, **(B)** IL-1β, **(C)** IL-6, **(D)** IL-13 and **(E)** MCP-1. (The figure shows the sum of 3 independent experiments, n=3 per group and for each mediator; *p<0.05; **p<0.01; ***p<0.001; N.D, Not detected; One Way ANOVA test).

Previous reports have shown that L.m induces the *de novo* synthesis of different cytokines and chemokines (16, 33). To further corroborate the MC response to L.m we determined the production of TNF-α, IL-1β, IL-6, IL-13 and MCP-1. We stimulated BMMC with different MOI of L.m and at 24 h we determined the levels of each mediator. We found that BMMC produced TNF-α with MOI 1:1, 10:1 and 100:1 of L.m (**Figure 2A**). While IL-1β production was only detected in BMMC incubated with MOI 100:1 of L.m (**Figure 2B**). Similarly, we found that this bacterium induced IL-6 (**Figure 2C**) and IL-13 (**Figure 2D**) only with MOI 100:1 of L.m in BMMC. While MCP-1 was induced with MOI 10:1 and 100:1 of L.m in BMMC (**Figure 2E**). Together, these results indicate that L.m induces *de novo* synthesis of cytokines and chemokines.

### *Listeria monocytogenes* Induces the Activation of Signaling Pathways Associated With TLR Activation on Mast Cells

**Figure 3:**
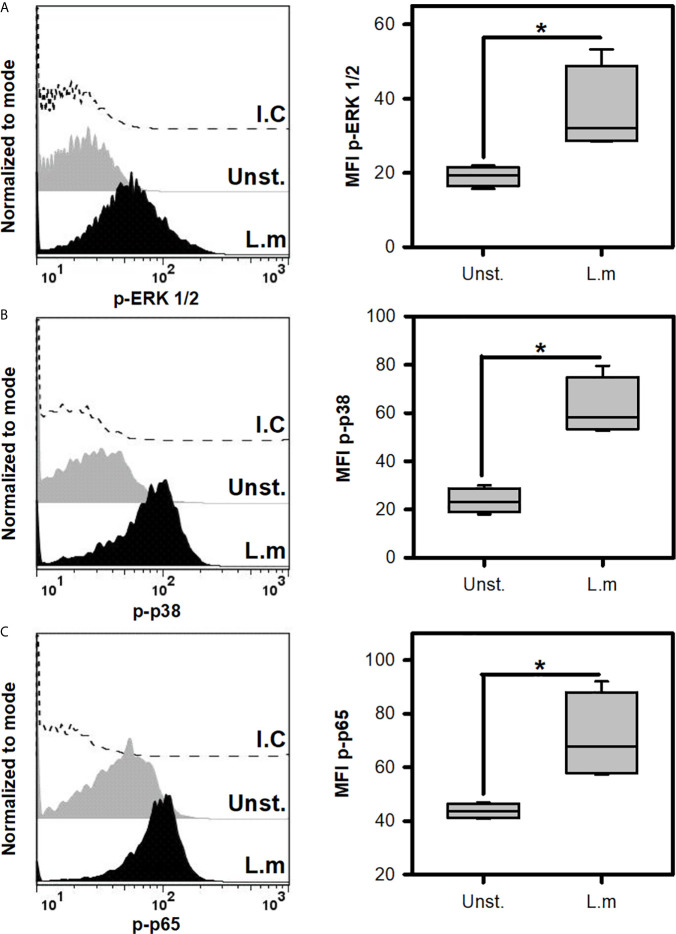
*Listeria monocytogenes* induce the phosphorylation of cell signaling proteins involved in TLR activation in mast cells. 2.5x10^5^ BMMC/0.25 mL of complete medium were stimulated with L.m at MOI 100:1 for different times to determine the phosphorylation of **(A)** ERK 1/2 at 15 minutes, **(B)** p38 at 60 minutes and **(C)** p65 at 30 minutes by flow cytometry. The graphs show the median fluorescence intensity (MFI). (Sum of 4 independent experiments, n=4 per group and for each phospho-protein. *p<0.05; Mann-Whitney test).

Since L.m promoted the activation of BMMC, we decided to evaluate if this was associated with the induction of some intracellular signals that are associated with TLR receptors (22). To this end, we incubated BMMC with L.m (MOI 100:1) and determined the phosphorylation of some signaling proteins by flow cytometry. Initially we evaluated the phosphorylation of ERK 1/2 (**Figure 3A**) and p38 (**Figure 3B**), molecules belonging to the MAPK signaling pathway and which have been related to the production of pro-inflammatory cytokines in macrophages infected with L.m (34, 35). Interestingly, we found that L.m induced phosphorylation of both signaling proteins in BMMC (**Figures 3A, B**). An important transcription factor in the production of pro-inflammatory cytokines and chemokines by macrophages infected with L.m is NF-κB (34). Therefore, we determined whether this transcription factor was activated in BMMC in response to L.m, by evaluating the phosphorylation of p65 subunit. Interestingly, we found that L.m induced phosphorylation of p65 in BMMC (**Figure 3C**). Together, these results indicate that L.m induces the activation in BMMC of cell signaling molecules that are associated with TLR activation.

### TLR2 Is Not Involved in Mast Cell Degranulation, ROS Release, Endocytosis and *Listeria monocytogenes* Clearance

**Figure 4:**
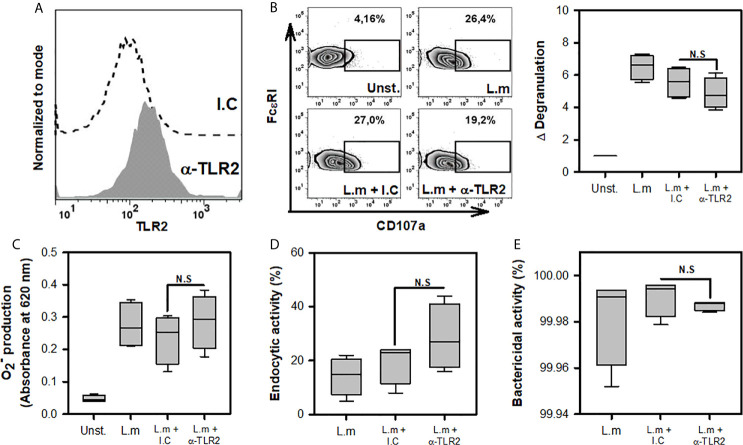
Mast cell degranulation, ROS production, endocytosis and clearance of Listeria monocytogenes are TLR2-independent. **(A)**. 2.5x10^5^ BMMC were marked with anti-TLR2-biotin (1 μg/0.1 mL) or biotinylated isotype control (I.C) (1 μg/0.1 mL) and stained with streptavidin-APC. Staining was evaluated by flow cytometry. **(B)** 2.5x10^5^ BMMC/0.25 mL of complete medium were preincubated with anti-TLR2 for 30 minutes and then were stimulated with L.m (MOI 100:1) for 90 minutes. BMMC degranulation was determined by flow cytometry. Left panel shows representative zebra-plots. Right panel shows the fold change in the percentage of FcϵRI+/CD107a+ cells with respect to unstimulated MC (sum of 4 independent experiments, n=4 per group; N.S, Not Significance; Kruskal-Wallis test). **(C)** 2.5x10^5^ BMMC/0.25 mL of complete medium were preincubated with anti-TLR2 for 30 minutes. Afterwards, were stimulated with L.m (MOI 100:1) for 2 h. Then, the determination was performed by the NBT reduction assay (sum of 4 independent experiments, n=4 per group; N.S, Not Significance; Kruskal-Wallis test). **(D, E)** 2.5x10^5^ BMMC/0.25 mL of complete medium were preincubated with anti-TLR2 for 30 minutes. Afterwards, were stimulated with L.m (MOI 100:1) for 2 h. Then the ${\text{O}}_{2}^{-}$intracellular bacteria were quantified by CFU assay. **(D)** Graphs show the percentage of endocytic activity (CFU at 0 h/MOI added x 100). **(E)** Graphs show the percentage of bactericidal activity (100-(CFU at 24 h/CFU at 0 h) x 100). (sum of 4 independent experiments, n=4 per group; N.S, Not Significance; Kruskal-Wallis test).

One of the main receptors found in various cells of the innate immune response that recognizes different components of the cell wall of Gram-positive bacteria, such as L.m, is TLR2 (20). The importance of macrophage TLR2 for phagocytosis and ROS production during infection with L.m has been demonstrated previously (36). Furthermore, BMMC TLR2 is known to participate in degranulation upon stimulation with peptidoglycan (PGN) (26). Based on these findings, we decided to evaluate whether TLR2 is involved in recognizing L.m and promoting the activation mechanisms in MC. Because some studies have suggested that human MCs do not express TLR (37), we evaluated the expression of this receptor on the surface of BMMC by flow cytometry. As reported previously (18, 22), this receptor was present in BMMC (**Figure 4A**). Afterwards, we pre-incubated BMMC with anti-TLR2 or isotype control for 30 minutes and then added to L.m (MOI 100:1). Then again, we evaluated cell degranulation through CD107a expression, ROS production with ${\text{O}}_{2}^{-}$ detection, endocytic and bactericidal activity with intracellular CFU. We found that TLR2 blockade did not affect degranulation (**Figure 4B**), the levels of ${\text{O}}_{2}^{-}$ produced (**Figure 4C**). We did not observe any change in the endocytic or bactericidal activity of BMMC incubated with L.m when TLR2 was blocked (**Figures 4D, E**). Altogether, our results indicate that MC TLR2 is not involved in the development of degranulation, ROS production, endocytosis or bacteria clearance during L.m infection.

### Mast Cell TLR2 Participates in IL-6 and IL-13 Production During *Listeria monocytogenes* Infection

**Figure 5:**
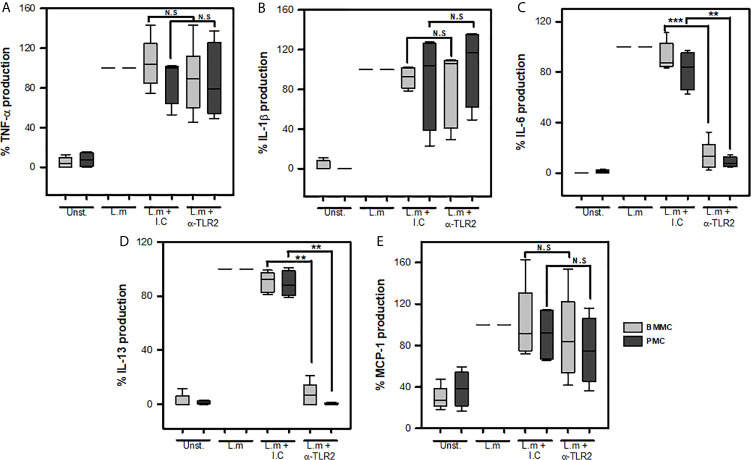
Mast cells TLR2 is required for IL-6 and IL-13 production in response to *Listeria monocytogenes*. 2.5x10^5^ BMMC or 2.5x10^5^ PMC in 0.25 mL of complete medium were preincubated with anti-TLR2 for 30 minutes and then were stimulated with L.m (MOI 100:1) for 24 h. Mediators levels were evaluated in culture supernatants by ELISA. **(A)** TNF-α, **(B)** IL-1β, **(C)** IL-6, **(D)** IL-13 and **(E)** MCP-1. Data are expressed as percentage of cytokine production, considering as 100% the production induced for L.m only. (Sum of 6 independent experiments, n=6 per group and for each mediator for BMMC (light grey boxes); sum of 4 independent experiments, n=4 per group and for each mediator for PMC (dark grey boxes); N.D, Not detected; N.S, Not Significance; **p<0.01, ***p<0.001; Kruskal-Wallis test).

Previous studies have shown the importance of macrophage TLR2 activation for the secretion of IL-1β, IL-6, TNF-α, IFN-β during the infection with L.m (38, 39). Furthermore, MyD88-deficient BMMC are reduced in their ability to produce IL-6 and MCP-1 in response to L.m implying a role of TLR in MC response (16). Therefore, we decided to investigate whether this MC TLR2 could mediate *de novo* synthesis of the cytokines TNF-α, IL-1β, IL-6, IL-13 and MCP-1 in two different sources of MCs: peritoneal MCs (PMC) and bone-marrow-derived MCs (BMMC). To this end, we pre-incubated MCs with 200 ng/mL anti-TLR2, a dose that efficiently blocks the interaction of TLR-2 and its natural ligand (**Supplementary Figure 5**), or its respective isotype control for 30 minutes and then stimulated with L.m MOI 100:1 for 24 h. We found that blocking TLR2 in both PMC and BMMC did not affect the levels of TNF-α (**Figure 5A**), IL-1β (**Figure 5B**) or MCP-1 (**Figure 5E**). However, we observed that blocking this receptor significantly reduced the levels of IL-6 (**Figure 5C**) and IL-13 (**Figure 5D**) in both PMC and BMMC. These results show that MC TLR2 does not participate in the production of TNF-α, IL-1β; and MCP-1; but regulates IL-6 and IL-13 production during L.m infection.

**Figure 6:**
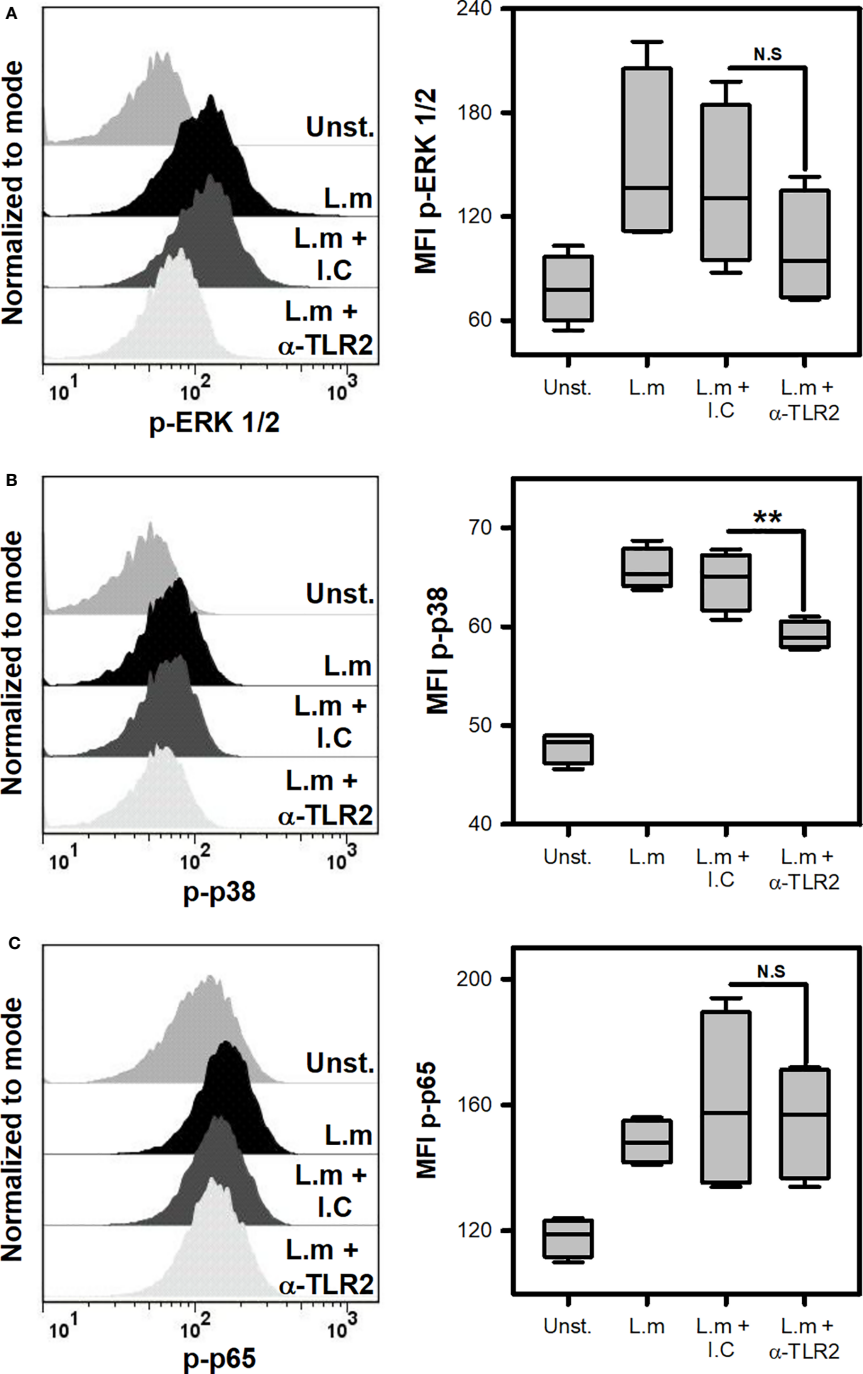
Mast cells TLR2 regulates p38 activation in response to *Listeria monocytogenes*. 2.5x10^5^ BMMC/0.25 mL of complete medium were preincubated with anti-TLR2 for 30 minutes and then were stimulated with L.m (MOI 100:1) for different times to determine the phosphorylation of **(A)** ERK 1/2 at 15 minutes, **(B)** p38 at 60 minutes and **(C)** p65 at 30 minutes by flow cytometry. The graphs show the median fluorescence intensity (MFI). (The figure shows the sum of 4 independent experiments, n=4 per group and for each phospho-protein. **p<0.01; N.S, Not Significance; Kruskal-Wallis test).

To discern which cell signaling pathway was regulated in MC by TLR2 during L.m infection, p38, ERK and p65 activation was evaluated after incubating MCs with anti-TLR2. Interestingly, we noticed that p38 phosphorylation was inhibited when TLR-2 was blocked, while ERK and p65 phosphorylation were not affected (**Figures 6A–C**). This result showed that p38 cell signaling is regulated by TLR2 in MCs during L.m infection.

**Figure 7:**
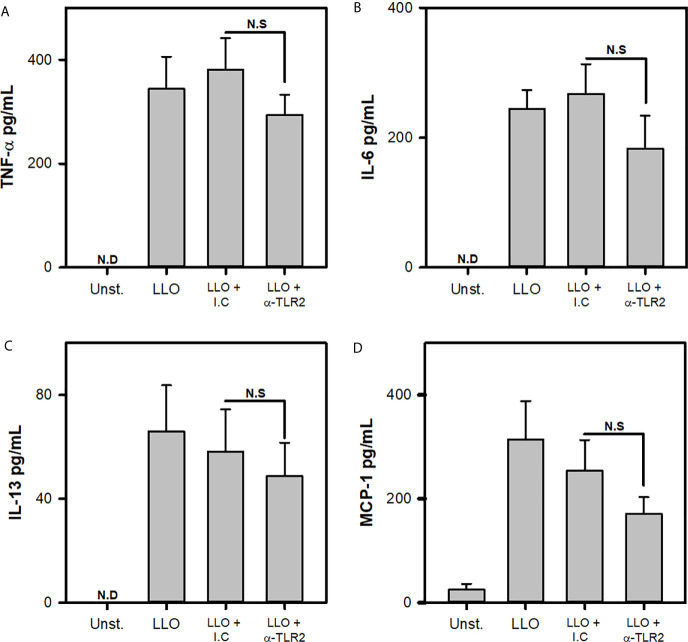
Cytokine production by mast cells in response to Listeriolysin-O is independent of TLR2. 2.5x10^5^ BMMC/0.25 mL of complete medium were preincubated with anti-TLR2 for 30 minutes and then were stimulated with 1000 ng/mL of Listeriolysin-O (LLO) for 24 h. Mediators levels were evaluated in culture supernatants by ELISA. **(A)** TNF-α, **(B)** IL-6, **(C)** IL-13 and **(D)** MCP-1. (Sum of 4 independent experiments, n=4 per group and for each mediator; N.D, Not detected; N.S, Not Significance; One Way ANOVA test).

Finally, previous reports indicate that L.m soluble products, like listeriolysin O, can activate MCs through increasing intracellular Ca^2+^ by altering endoplasmic reticulum independently of MCs receptors (17). To evaluate whether MC TLR2 was involved in LLO mediated activation, BMMCs were incubated with blocking antibodies to TLR-2 and stimulated with 1000 ng/mL LLO, a dose that did not induced MC apoptosis nor necrosis, but induced cytokine production (**Supplementary Figure 2** and **3**). We noticed that MC released similar amounts of TNF-α, IL-6, IL-13 and MCP-1 in response to LLO independently of TLR2 (**Figures 7A–D**). These results indicated that TLR2 was not necessary for MC activation by LLO.

**Figure 8:**
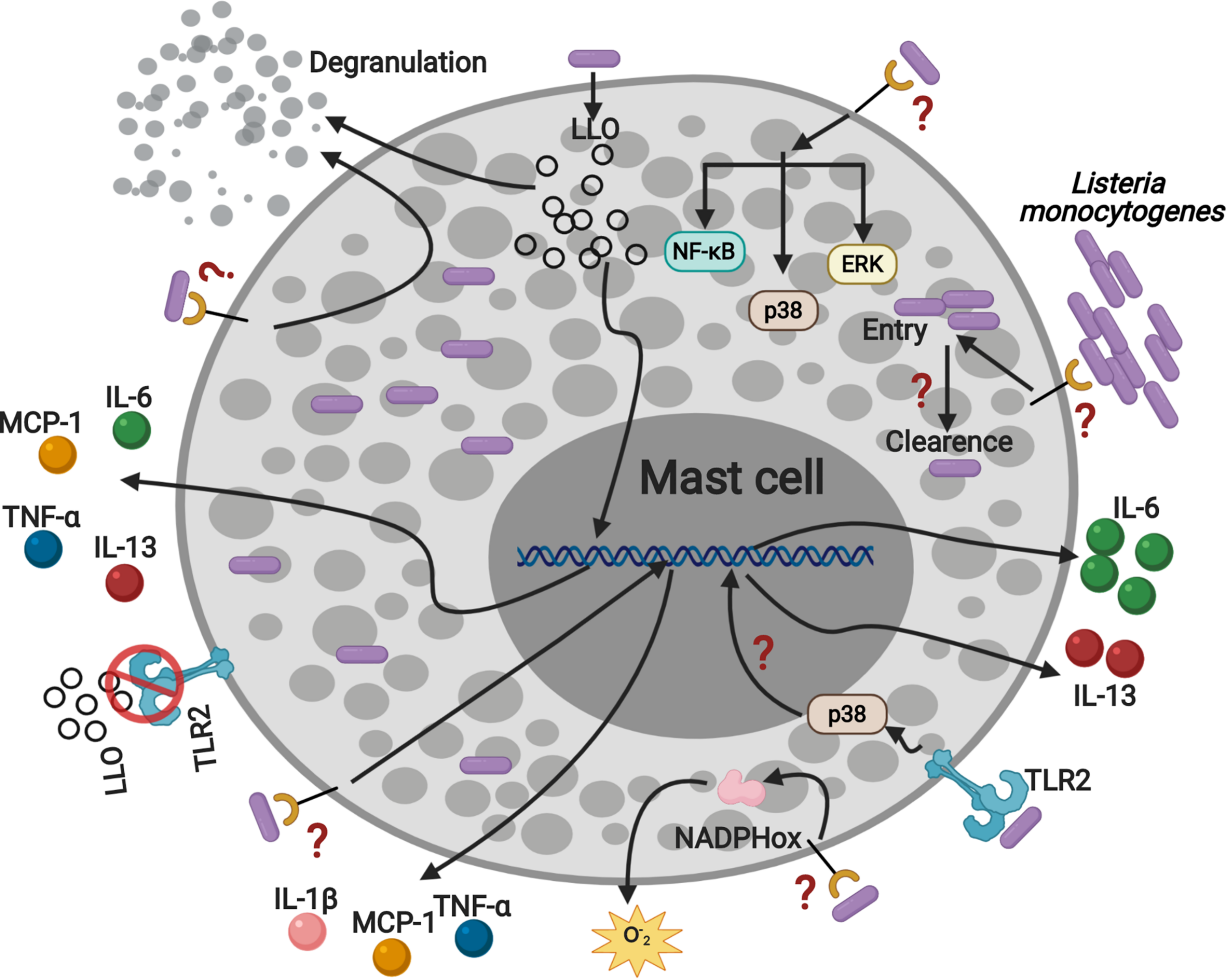
*Listeria monocytogenes* activates mast cell through TLR2 inducing p38 activation, IL-6 and IL-13 production. MC are infected by L.m and they can eliminate the intracellular bacterial load. In addition, L.m infection activates several signaling proteins: ERK, p38 and NF-κB, which could drive MC to degranulate, ROS and cytokines production. Furthermore, the recognition of L.m by TLR2 promotes p38 activation and the production of IL-6 and IL-13. In addition, LLO induced the production of TNF-α, IL-6, IL-13 and MCP-1 independently of TLR2, indicating that other mechanisms or mast cell receptors are regulating the interaction with L.m. This figure was created with BioRender.com.

Collectively, our data show that MC activation by TLR2 during L.m infection regulates IL-6 and IL-13 production and suggest that other receptors could be involved in mediating other mechanisms of MC activation (**Figure 8**).

## Discussion

MC are cells of the innate immune system that are key to the control of diverse infectious processes, since they promote the recruitment of other effector cells, through the release of cytokines and chemokines (2)⁠. In experimental murine listeriosis model, MC are crucial in controlling the infection by promoting recruitment of neutrophils to the site of infection (10). Therefore, this suggests that MC can recognize L.m and get activated. However, little is known about the MC receptors involved in recognizing the bacteria and whether these interactions mediate MC activation.

In this study, we confirmed that L.m activates MC by inducing cell degranulation and ROS production. In addition, L.m infects MC, but they control the intracellular bacterial load. Moreover, MC produce different cytokines in response to L.m. We also provide evidence that L.m promotes the activation of some intracellular signaling pathways in MC that are involved in TLR activation. Furthermore, we demonstrate that TLR2 is not involved in cell degranulation, ROS release or in the endocytosis and clearance of L.m. Similarly, MC TLR2 does not participate in the production of the inflammatory cytokines TNF-α, IL-1β and MCP-1; but it is crucial for the production of IL-6 and IL-13 in response to L.m infection.

MAPK pathway and NF-kB has been reported to be activated on macrophages after infection with L.m, and their expression are related with the production of different cytokines (34, 40, 41). In the case of MC, MAPK is induced in response to hemolysin- producing *Escherichia coli* (42), streptococcal exotoxin streptolysin O (43), and *E. coli* LPS (44); and NF-kB in response to *E. coli* LPS (29) and *Candida albicans* (45)*⁠*. Here, we show for the first time that L.m induces the activation of MAPK molecules (ERK and p38) and NF-kB in BMMC, indicating that these signaling pathways are active in these cells and could promote the cytokine and MCP-1 production.

TLR2 is one of the main receptors expressed in cells of the innate immune response. TLR2 is one of the main receptors expressed in cells of the innate immune response. Unlike TLR4, which recognizes ligands such as lipopolysaccharide (LPS) and mannuronic acid polymers from Gram-negative bacteria, some viral components and Damage-associated molecular patterns (DAMP) (46). TLR2 recognizes different ligands including diacyl-lipopeptides, lipoarabinomannan, lipoproteins, lipoteichoic acid (LTA), peptidoglycan (PGN), porins, phospholipomannan and zymosan (20). Although TLR4 also recognizes ligands from Gram-positive bacteria such as LTA (46) or LLO from L.m (47), TLR2 seems to play a more important role in the recognition of Gram-positive bacteria, such as L. m (20) and is expressed on MC, as shown here and by others (29, 48, 49). Interestingly, TLR2-deficient mice are susceptible to L.m infection (28). Although TLR2 has been involved in MC degranulation against *S. aureus* PGN (26), L.m seems not to be inducing this mechanism through TLR2. Therefore, our findings suggest that TLR2-independent interactions promote MC degranulation against L.m.

ROS production in L.m infected MC is associated with the release of MC extracellular traps (MCET), through a mechanism dependent on NADPH oxidase, MCET contribute to L.m extracellular clearance (18). Moreover, ROS production also promotes the intracellular clearance of internalized *E. coli* in MC endosomes (50). Furthermore, TLR2 promotes in L.m infected macrophages to the release of mitochondrial ROS associated with TNF-α, IL-1β and IL-6 synthesis *via* NF-kB and MAPK activation (36). However, our findings suggest that ROS release by L.m infected MC is not associated with TLR2- interactions.

Despite L.m endocytosis by macrophages has been shown be dependent of PI3K-AKT-Rac1 signaling pathway induced by TLR2 (23), we did not find any involvement of TLR2 in L.m endocytosis by MC. One possibility is that L.m entry does not involve TLR2 in MC. Additionally, we also noticed that MC TLR2 did not participate in L.m intracellular clearance, contrary to what is observed in TLR2-deficient MC infected with *F. tularensis* (24). This suggests that different signals may promote the activation of intrinsic microbicidal mechanisms in MC or that L.m virulence factors may promote the permanence of this bacterium within MC (16).

MC release a wide variety of cytokines and chemokines in response to L.m, including TNF-α, IL-1β, IL-6, IL-13 and MCP-1 (15, 16, 33). Interestingly, TNF-α or TNF-receptor deficient mice are highly susceptible to L.m infection (51–53). This could be associated with the TNF-α exacerbation of intracellular killing of L.m by macrophages through ROS and nitric oxide production (54, 55). On the other hand, antibody-mediated IL-1 receptor blockade affects neutrophil recruitment and macrophage activation in L.m-infected mice (56), which coincides with an increase in bacterial load in the spleen and liver (57). Similarly, mice deficient in MCP-1 are more susceptible to L.m infection, which correlates with poor recruitment of inflammatory monocytes (58). While IL-6 deficient mice are unable to mobilize neutrophils from the bone marrow into the blood circulation, making them highly susceptible to L.m infection (59). Finally, administration of IL-13 to mice infected with L.m favors infection control, which coincides with enhanced NK cells activation and an increase in serum IL-12 concentration (60). Therefore, these mediators produced by MC may contribute significantly to host defense against L. m infection.

In contrast to previous reports where it has been shown that TLR2-deficient MC exhibit a reduced production of TNF-α in response to *S. aureus* PGN (29) and *S. equi* (61), we found TNF-α production was independent to TLR2 signals. A possibility for this difference could rely on the ability of L.m to release the virulent factor LLO. Previous studies, showed that LLO increases intracellular calcium levels in MC, leading to activation of NFAT, a transcription factor that promotes TNF-α synthesis (17). Furthermore, we observed that BMMC produced significant amount of TNF-α after being stimulated with LLO (**Supplementary Figure 3**), and this production was not affected when TLR2 was blocked (**Figure 7A**). This suggest LLO-activated signaling pathway is more relevant than TLR2 for the production of TNF-α in MC.

Similarly, TLR2 was not involved in IL-1β production by L.m infected MC. This coincides with findings in MyD88 or TLR2/4 -deficient MC infected with L.m (33). IL-1β production in L.m-infected macrophages depends on both the recognition of L.m lipoproteins by TLR2 and the recognition of L.m PGN fragments by NOD1/NOD2 receptors intracellularly. In both cases, pro-IL-1β transcription through NF-kB is induced (62, 63). Therefore, we suggest that TLR2 is not mediating IL-1β production by L.m infected MC and that PGN of this bacterium may induce its production through their recognition by NOD1/NOD2. Furthermore, other interactions could mediate the production of this cytokine, such as integrin α1β2 (CD49b/CD29), which has been shown partially involved in IL-1β production by L.m stimulated MC (64).

We observed that TLR2 was not involved in MCP-1 production by L.m infected MC. Interestingly, TLR2 is important for the production of this chemokine by *S. equi* infected MC (61)⁠. This suggests that other PRR are involved in the production of MCP-1 in response to L.m. MCP-1 production is dependent on NF-κB activation (65). Therefore, different signaling pathways that converge in NF-κB may be involved in its production. The recognition of L.m PGN by NOD1/NOD2 receptors would contribute to the activation of NF-κB and thus the induction of MCP-1. Interestingly, MCP-1 production in MC infected with L.m, depends partially on the signaling carried by MyD88, E-cadherin-IntA interaction and LLO (16)⁠.

However, we observed that TLR2 participates in the production of IL-6 and IL-13 in MC infected with L.m. In fact, TLR2-deficient MC exhibit a reduced production of IL-6 in response to *S. aureus* PGN (26) and *S. equi* (61); and MyD88-deficient MC stimulated with *S. aureus* PGN and L.m are unable to produce IL-6 (16). Moreover, IL-13 production is TLR2-dependent in MC stimulated with bacterial pathogens such as *S. equi* (61) or with peptidoglycan from *S. aureus* (26). Therefore, we suggest that MC TLR2 may recognize the cell wall components of L.m and thus promote IL-6 and IL-13 synthesis. IL-6 expression is generally associated with NF-κB, MAPK/AP-1, C/EBP, CREB and PI3K/AKT activation (66–69) and interestingly, TLR2-MyD88 signaling leads to activation of NF-κB, MAPK/AP-1, PI3K/AKT (22, 70). Our results indicate L.m is inducing NF-κB, ERK and p38 activation. Interestingly, p38 was the only cell signaling pathway modulated by TLR2 which could be involved in IL-6 and IL-13 production, as has been described in BMMC stimulated with IL-33 (71). On the other hand, a recent study demonstrated that L.m induces ERK 1/2 phosphorylation in human epithelial Caco-2 cells, mediated mainly by LLO binding to cholesterol and subsequent pore formation. Interestingly, this effect correlates with the ability of L.m to invade and replicate into fibroblasts and macrophages (72). Therefore, we suggest that ERK 1/2 activation in L.m-stimulated MC may be LLO-dependent. However, it is unclear whether L.m takes advantage of this signaling pathway in MC to invade and replicate in them beyond promoting the induction of proinflammatory cytokines.

Interestingly, we found that LLO induced the release of IL-6 and IL-13 by MC, without requiring TLR2 signaling. This shows that multiple L.m pathogen-associated molecular patterns (PAMPs) lead to IL-6 and IL-13 production, indicating a vital role of these cytokines in the host immune response to L.m, and suggests that LLO could be recognized by MC through other receptors, with TLR4 being a potential candidate (47, 73).

IL-13 together with IL-4, IL-5 and IL-10 belong to the group of cytokines of the type 2 response profile, since they promote the induction of the humoral immune response by favoring the production of antibodies by B cells, diminish the cellular immune response and lead to an anti-inflammatory environment (74, 75). The type response profile 2 cytokines and other mediators produced by MC are able to direct, amplify and perpetuate the Th2 immune response in allergy, helminthic infection and in oral immunization models (76–80). Interestingly type 2 response has been recently associated as a relevant mechanism during bacterial infection, in particular during the skin infection with *S. aureus*, by favoring IgE production and effector mechanisms regulated by MC (81). Our results suggest that MC TLR2 could have a relevant role during the innate immune response to bacteria and promote an environment that favors type-2 immune response. However, it is unclear whether this response could be elicited by L.m. Our findings suggest that IL-13 production by MC responds to different L.m-activated signals, some independent of TLR2 (as is the case with LLO). This is surprising since previous studies have shown that LLO inhibits Th2-mediated reactions in murine models of allergic rhinitis (82). Thus, the functional role of IL-13 produced by MC in the context of L.m infection needs to be further explored.

In conclusion, our results show that L.m induces the activation of signaling pathways in mast cells, mainly related to cytokines synthesis. In addition, TLR2 participates in IL-6 and IL-13 production and p38 activation. While TNF-α, IL-1β, MCP-1 production, ROS release, cell degranulation, the endocytic and bactericidal activity of mast cells, as well as ERK and NF-κB activation are TLR2-independent mechanisms. Therefore, we demonstrate that mast cell TLR2 plays a crucial role in regulating the synthesis of IL-6 and IL-13 during *Listeria monocytogenes* infection in MC.

## Data Availability Statement

The original contributions presented in the study are included in the article/**Supplementary Material**. Further inquiries can be directed to the corresponding authors.

## Ethics Statement

The animal study was reviewed and approved by Research Ethics Committee of the ENCB, IPN.

## Author Contributions

Experimental design and analysis: RS-C, AA-D, GR-L, MC-N, YM-P, AG-S, VA-J, JY-P, RM-F, AD-F, SE-P, SP-T, AC-B, and RC-S. Experimental performing: RS-C, AA-D, GR-L, YM-P, and AG-S. Manuscript preparation: RS-C, GR-L, MC-N, VA-J, JY-P, RM-F, and RC-S. Funding: AC-B and RC-S. All authors contributed to the article and approved the submitted version.

## Funding

This research was supported by SIP, IPN (20210225), and Conacyt Ciencia Básica (258738).

## Conflict of Interest

The authors declare that the research was conducted in the absence of any commercial or financial relationships that could be construed as a potential conflict of interest.
