# Supplementary material for: TLR2 Regulates Mast Cell IL-6 and IL-13 Production During Listeria monocytogenes Infection
Source: Front Immunol. 2021 Jun 14;12:650779. doi: 10.3389/fimmu.2021.650779 (PMC8238461; doi:10.3389/fimmu.2021.650779)
Supplement: Supplementary file 1 [file DataSheet_1.pdf]

Supplementary Material

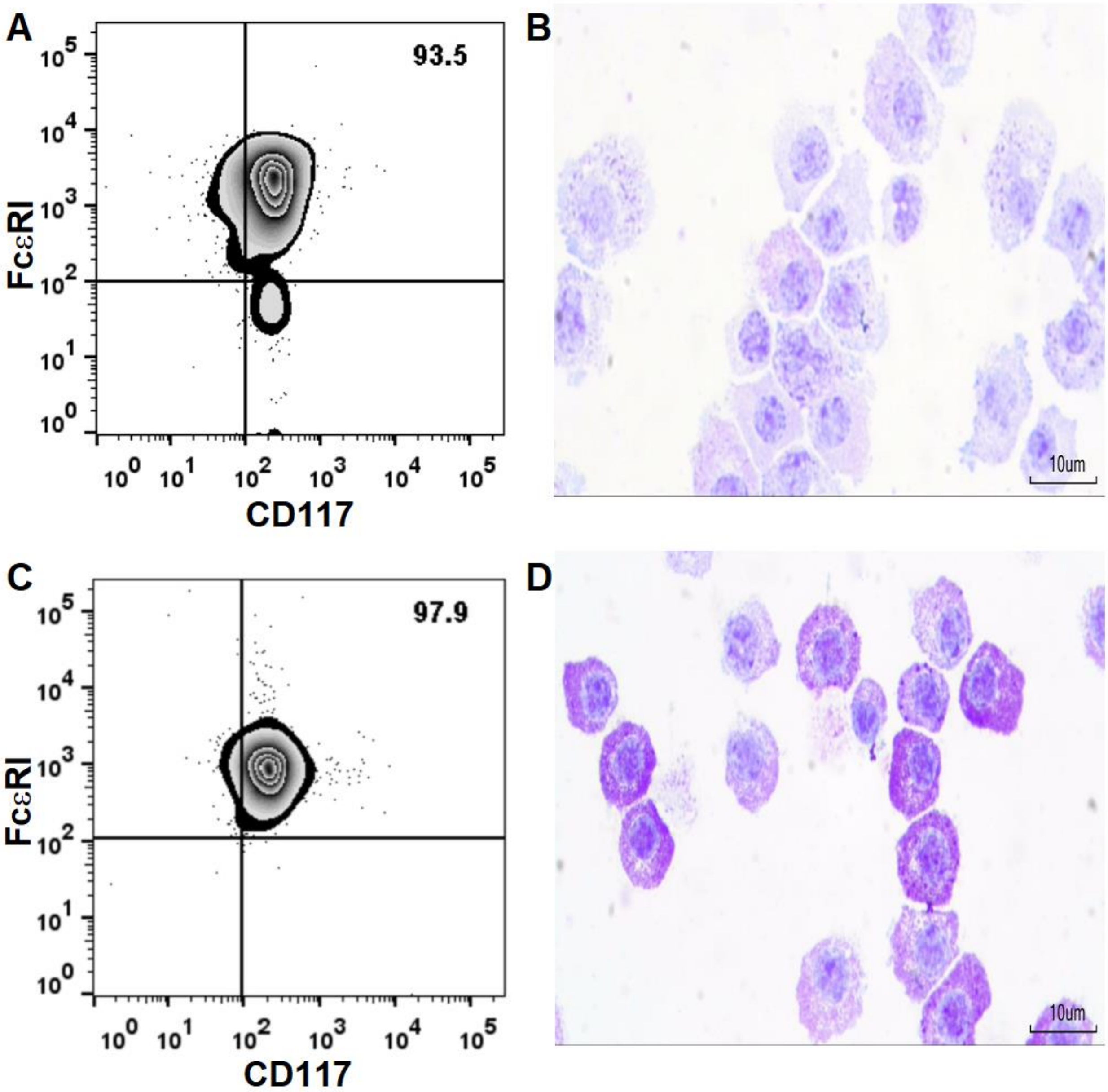

Supplementary Figure 1. Characterization of primary cultures of bone marrow and peritoneum-derived mast cells. Representative flow cytometry zebra plot of **(A)** bone marrow-derived mast cells (BMMC) and **(C)** peritoneum-derived mast cells (PMC). The percentage of FcεRI+CD117+ is shown. BMMC **(B)** and PMC **(D)** were stained with toluidine blue and analyzed by light microscopy. bar= 10 µm

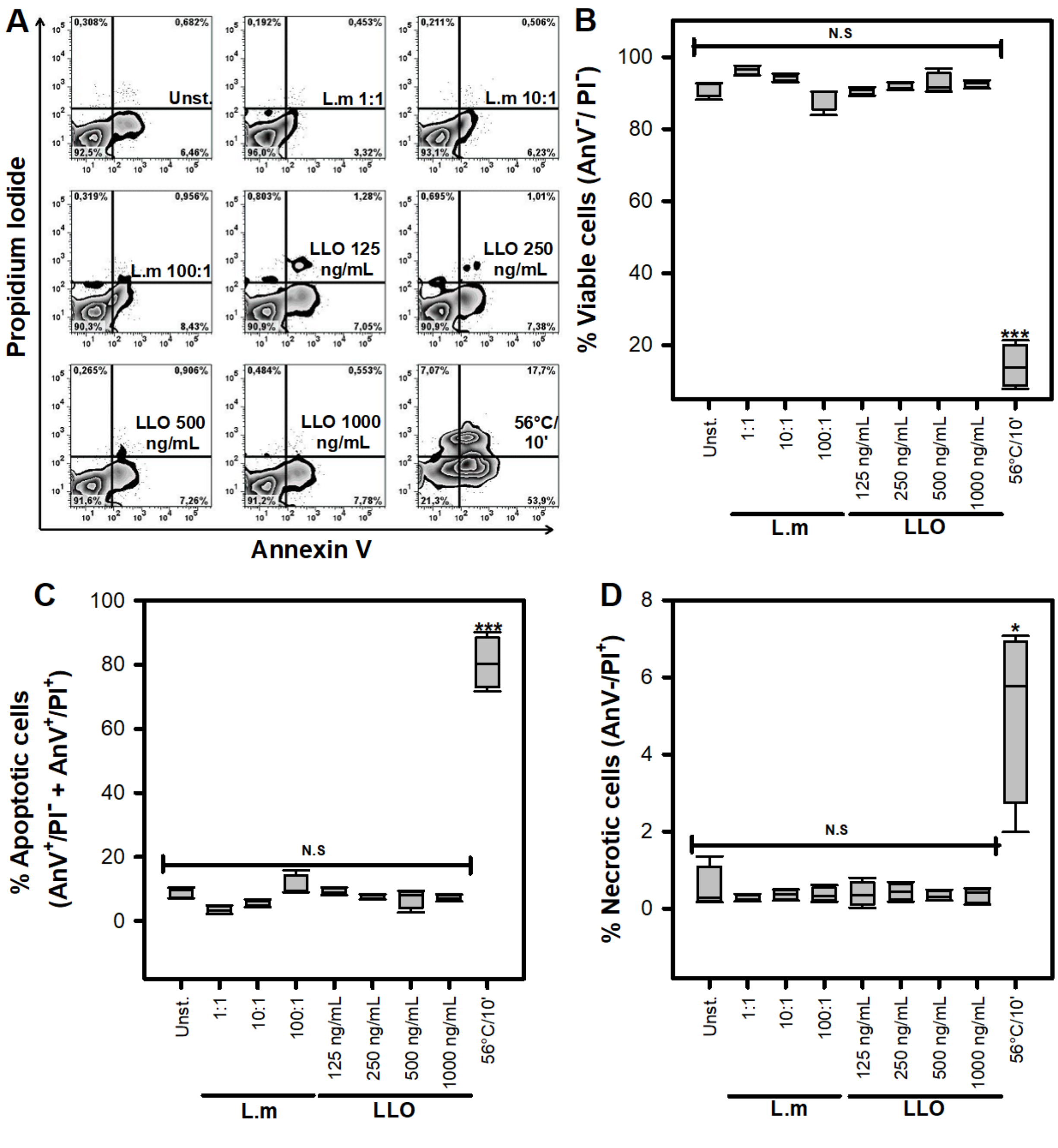

Supplementary Figure 2. Effect of *Listeria monocytogenes* and Listeriolysin-O on the viability of mast cells. $2x10^5$ BMMC were stimulated with L.m (MOI 1:1, 10:1 and 100:1) or LLO (125, 250, 500 and 1000 ng/mL) for 24 h. Cell viability was determined with Annexin V and propidium iodide (PI) staining and measured by flow cytometry. **(A)** Representative zebra-plots of BMMC stained with Annexin V and PI. **(B)** Percentage of viable cells (Annexin V-/PI-). **(C)** Percentage of total apoptotic cells represented as the sum of the percentage of early apoptotic cells (Annexin V+/PI-) and the percentage of late apoptotic cells (Annexin V+/PI+). **(D)** Percentage of necrotic cells (Annexin V-/PI-). (Sum of 4 independent experiments, n=4 per group; *p<0.05; ***p<0.001; **N.S=** Not Significance; comparisons were performed against unstimulated cells (Unst.). Kruskal-Wallis test).

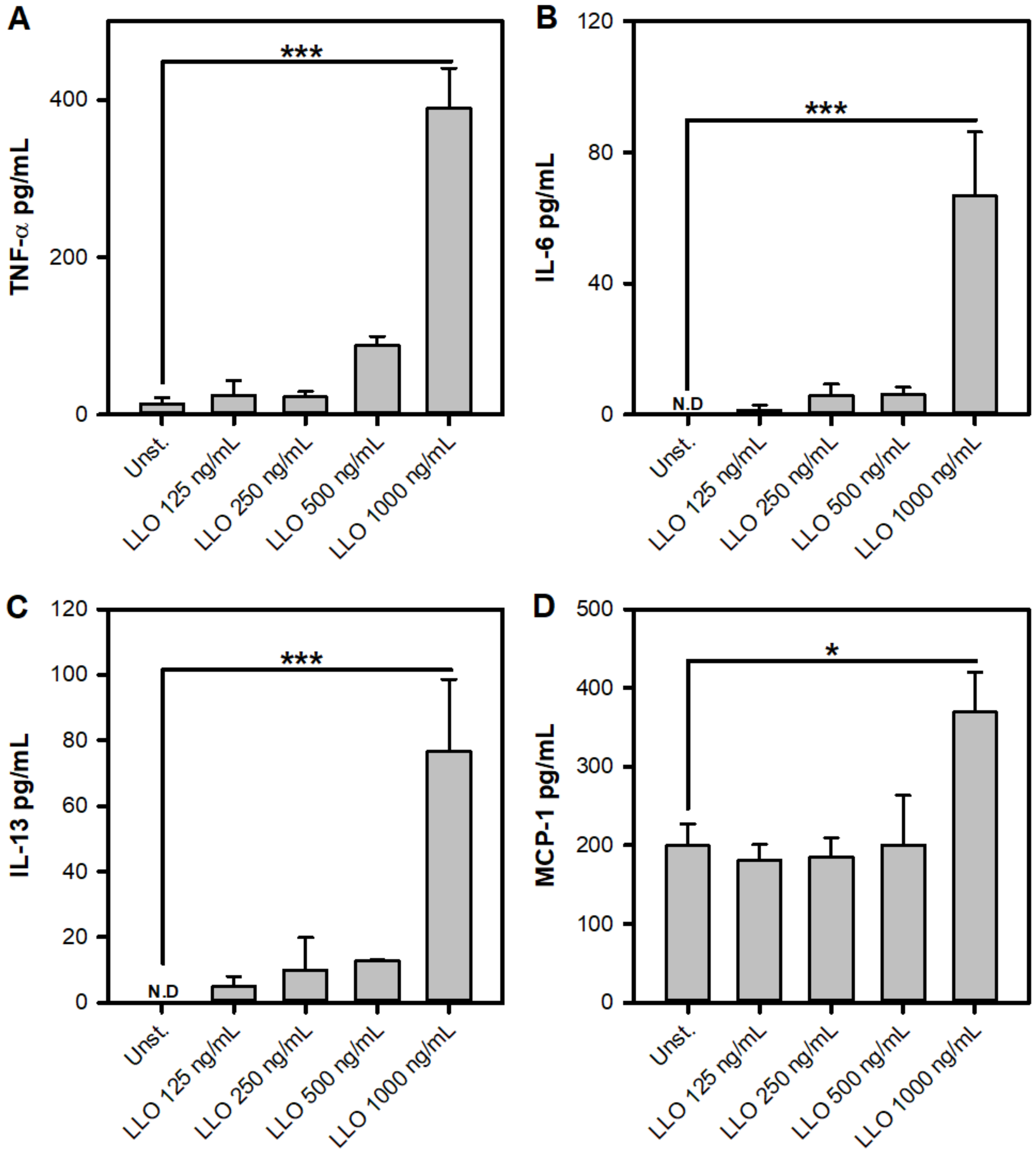

Supplementary Figure 3. Listeriolysin-O induces *de novo* synthesis of cytokines by mast cells. $2.5x10^5$ BMMC/ 0.25 mL of complete medium were stimulated with LLO for 24 h at the concentrations indicated. Cytokine levels were evaluated in culture supernatants by ELISA. **(A)** TNF-*α*, **(B)** IL-6, **(C)** IL-13 and **(D)** MCP-1. (Sum of 4 independent experiments, n=4 per group and for each mediator; *p<0.05; ***p<0.001; **N.D**= Not detected; One Way ANOVA test).

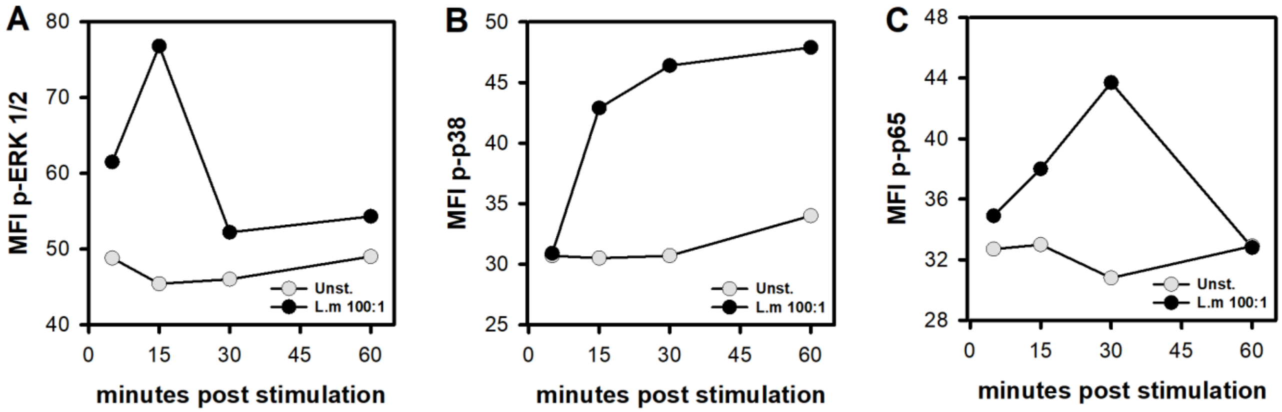

Supplementary Figure 4. Kinetic of protein phosphorylation in response to *Listeria monocytogenes* in mast cells. 2.5x10^5 BMMC/ 0.25 mL of complete medium were stimulated with L.m (MOI 100:1) for different times to determine the phosphorylation of **(A)** ERK 1/2, **(B)** p38 and **(C)** p65 by flow cytometry. The graphs show the median fluorescence intensity (MFI).

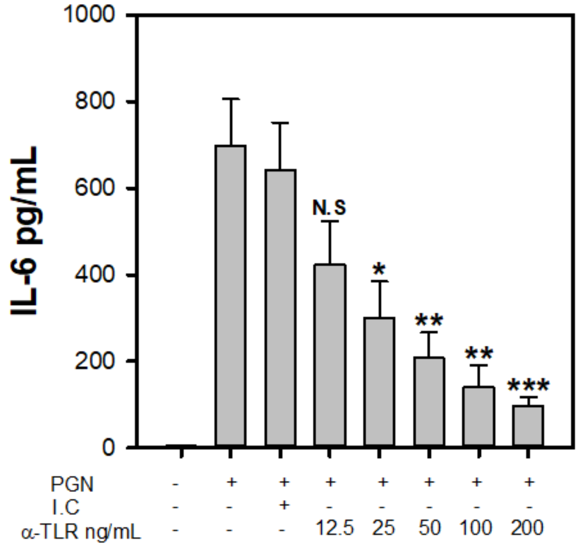

Supplementary Figure 5. Efficiency of antibody-mediated TLR2 inhibition. 2.5x10^5 BMMC/ 0.25 mL of complete medium were pre-incubated with anti-TLR2 at the concentrations indicated for 30 minutes and then were stimulated with *Staphylococcus aureus* peptidoglycan (PGN) at 10 µg/mL for 24 h. IL-6 level was evaluated in culture supernatants by ELISA. (Sum of 4 independent experiments, n=4 per group; *p<0.05; **p<0.01; ***p<0.001; **N.S**= Not Significance; comparisons were performed against cells stimulated with PGN and treated with Isotype Control (I.C). One Way ANOVA test).
